# Data supporting the morphological/topographical properties and the degradability on PET/PLA and PET/chitosan blends

**DOI:** 10.1016/j.dib.2019.104012

**Published:** 2019-05-24

**Authors:** D. Palma-Ramírez, A.M. Torres-Huerta, M.A. Domínguez-Crespo, D. Del Angel-López, A.I. Flores-Vela, D. de la Fuente

**Affiliations:** a Instituto Politécnico Nacional, CICATA-Altamira, Km. 14.5 Carretera Tampico-Puerto Industrial Altamira, 89600, Altamira, Tamps, Mexico; b Instituto Politécnico Nacional, Centro Mexicano para la Producción más Limpia (CMPL), Av. Acueducto s/n, la Laguna Ticomán, C.P. 07340, México City, Mexico; c Tecnológico de Monterrey, Escuela de Ingeniería y Ciencias, Ave Eugenio Garza Sada 2501, C.P. 64849, Monterrey N.L., Mexico; d Centro Nacional de Investigaciones Metalúrgicas, CENIM (CSIC), Av. Gregorio del Amo 8, 28040, Madrid, Spain

**Keywords:** PET/PLA, PET/Chitosan, SEM micrographs, AFM images, Roughness, Proposed degradation mechanism

## Abstract

These data display evidence of the fracture through the morphologies and the topographical features as well as roughness data of different ratios of R(recycled)-PET/PLA, PET(virgin)/PLA, PET(virgin)/Chitosan and R(recycled)-PET/chitosan. Also, data of the morphologies after degradation under accelerated weathering test and degradation mechanisms are revealed. The data supplement the article “Comparative assessment of miscibility and degradability on PET/PLA and PET/chitosan blends”.

**Table:** Specifications Table

Subject area	*Materials Science*
More specific subject area	*Polymers and Plastics*
Type of data	*Figure, Table and image (microscopy)*
How data was acquired	*Scanning Electron Microscopy (SEM) (SEM JEOL 6300 and JEOL-JSM-6500 F).* *Atomic force microscopy (AFM) (Nanosurf easyscan* 2 AFM*/STM).*
Data format	***Raw and analyzed***
Experimental factors	*The samples were dried at 40 °C for 24 h and then coated with an Au–Pd thin film. For SEM, samples were sputter coated with Au–Pd for 30 s on a Quorum Q150T ES sputter coater system.*
Experimental features	*Virgin or recycled PET/PLA, Virgin or recycled PET/Chitosan filaments were analyzed by their morphological and topographical features.*
Data source location	*Centro de Investigación en Ciencia Aplicada y Tecnología Avanzada (CICATA) IPN Unidad Altamira, Tamaulipas, México.* *CSIC-Centro Nacional de Investigaciones Metalúrgicas (CENIM), Madrid, Spain.*
Data accessibility	*Data are with the article.*
Related research article	*A.M. Torres-Huerta, D. Palma-Ramírez, M.A. Domínguez-Crespo, D. Del Angel-López, D. de la Fuente. Comparative assessment of miscibility and degradability on PET/PLA and PET/chitosan blends,* *European Polymer Journal 61 (2014) 285–299*[1]*.*

**Table:** 

**Value of the Data** • The data is valuable for informing researchers the topographical changes after blending the conventional PET or R-PET with either PLA or Chitosan biopolymers. • Data is valuable for comparing the type of fracture after tensile test between different ratio of R-PET/PLA, PET/PLA, PET/Chitosan and R-PET/chitosan. • The data will benefit to the studies related to the environment to understand how the morphological properties can be managed and increase the biodegradability in R-PET and R-PET systems with biopolymers. • The roughness data acquired from AFM measurements of PET/PLA, PET/PLA, PET/Chitosan and R-PET/chitosan are of additional value and will help to other studies to compare the topography and evaluate the changes on surface roughness according to the degradation time.

## 1 Data

One of the options to reduce the pollution problem derived of the long-lasting petroleum polymers use, such as poly(ethylenterephthalate) (PET), is to combine the mechanical, barrier and thermal properties of petroleum-based polymers with the biodegradability properties of renewable polymers: poly(lactic acid) (PLA) and chitosan [2], [3].

**Fig. 1:**
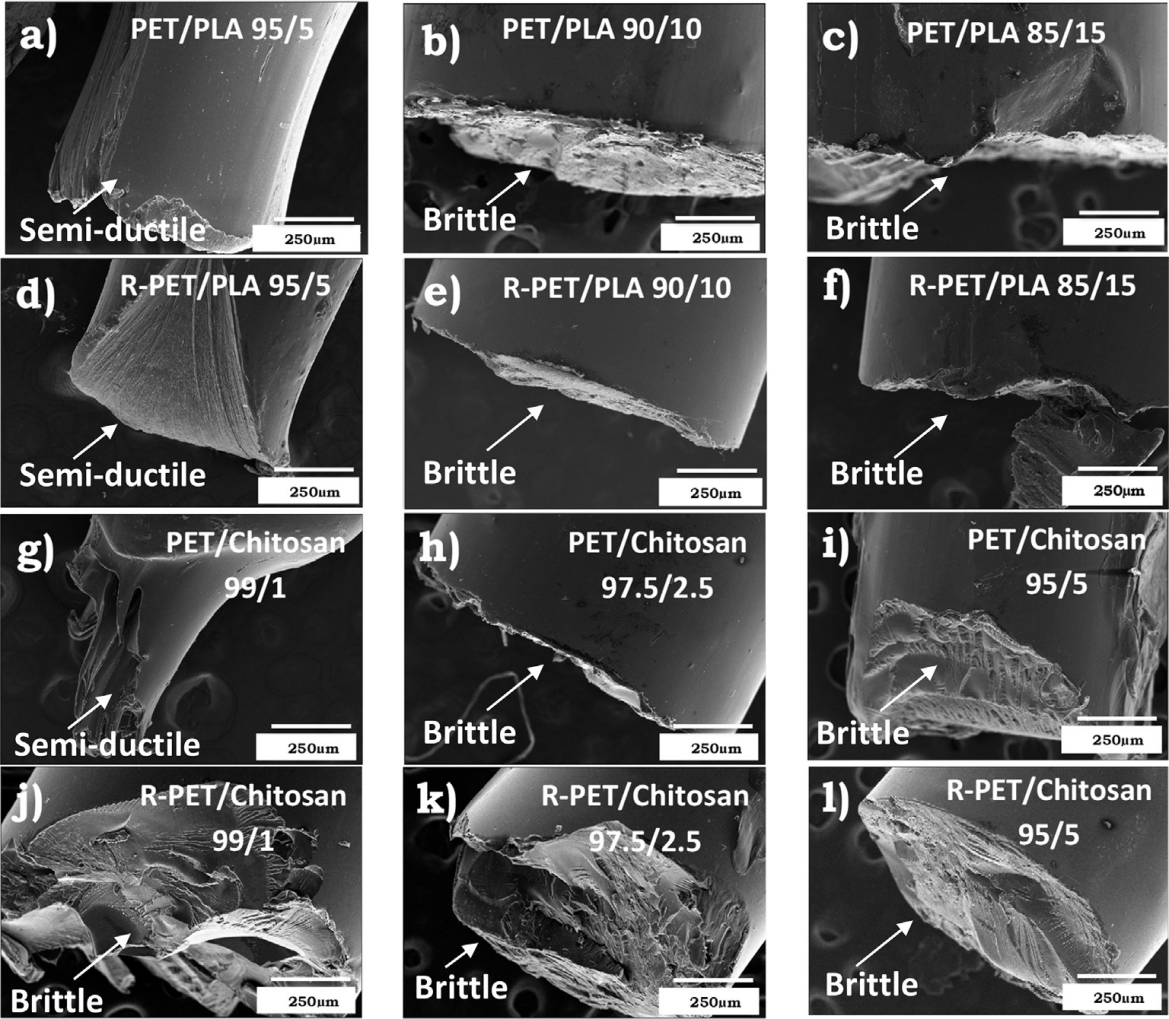
SEM micrographs of fractured a-c) PET/PLA, d-f) R-PET/PLA, g-i) PET/Chitosan and j-l) R-PET/chitosan filaments showing how the wt.% of addition of PLA and chitosan biopolymers modify the morphology of PET and R-PET, which is detected from the surface fractured.

The dataset of this work shows additional information related to the final morphology and topography of PET/PLA, R-PET/PLA, PET/Chitosan and R-PET/chitosan obtained by extrusion method. The filaments produced were fractured during the tensile test. The changes from fragile to ductile fracture with the addition of biopolymers can be distinguished by studying their morphologies. R-PET/PLA, PET/PLA, PET/Chitosan and R-PET/chitosan blends in different ratios were evaluated by Scanning Electron Microscopy (SEM). Fig. 1 shows the SEM micrographs corresponding to the PET or R-PET either with PLA (Fig. 1a–f) or chitosan (Fig. 1g–l) in different ratios.

**Fig. 2:**
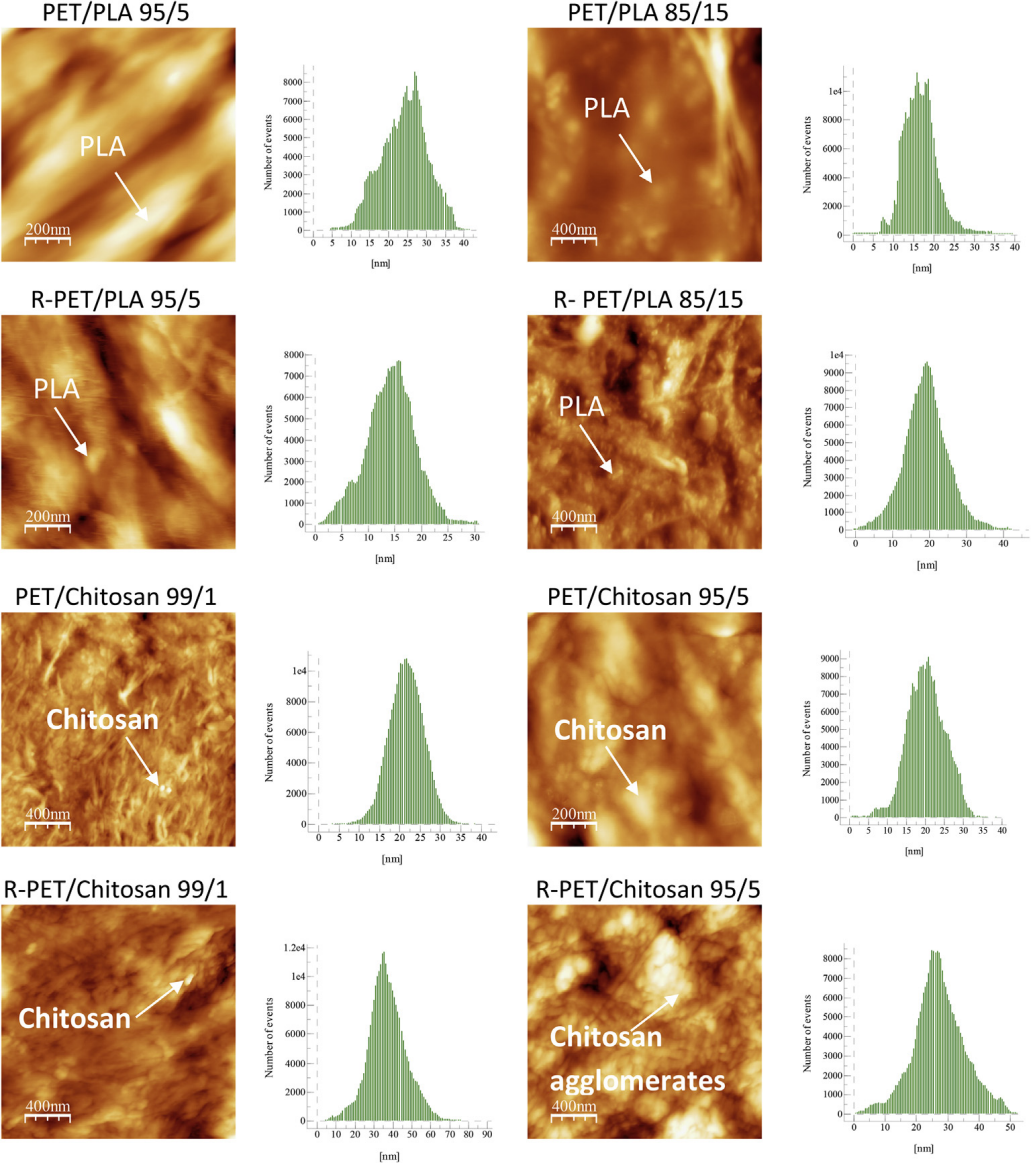
AFM micrographs of PET/PLA, R-PET/PLA, PET/Chitosan and R-PET/chitosan selected samples showing the topography and the incorporation of biopolymers particles as contrasted phase either individual or agglomerates into the polymeric matrixes.

The topography and the roughness value of these blends obtained by extrusion method, were studied by atomic force microscopy (AFM). Fig. 2 shows the phase contrast between PET, R-PET and biopolymers as well as the evolution of the topography in selected samples of PET/PLA, R-PET/PLA, PET/Chitosan and R-PET/chitosan. Table 1 contains the root-mean-square roughness (RMS) and the roughness average (Ra) acquired from AFM measurements.

**Table 1:** Roughness data obtained by atomic force microscopy (AFM) analysis.

Sample	RMS value (nm)	Ra value (nm)
PET/PLA 95/5	7.1233	5.7240
PET/PLA 85/15	9.6218	7.4755
R-PET/PLA 95/5	4.6615	3.6592
R-PET/PLA 85/15	6.8383	5.2365
PET/Chitosan 99/1	6.1008	4.4058
PET/Chitosan 95/5	5.7214	4.5222
R-PET/Chitosan 99/1	8.1459	6.3411
R-PET/Chitosan 95/5	6.0693	4.6352

**Fig. 3:**
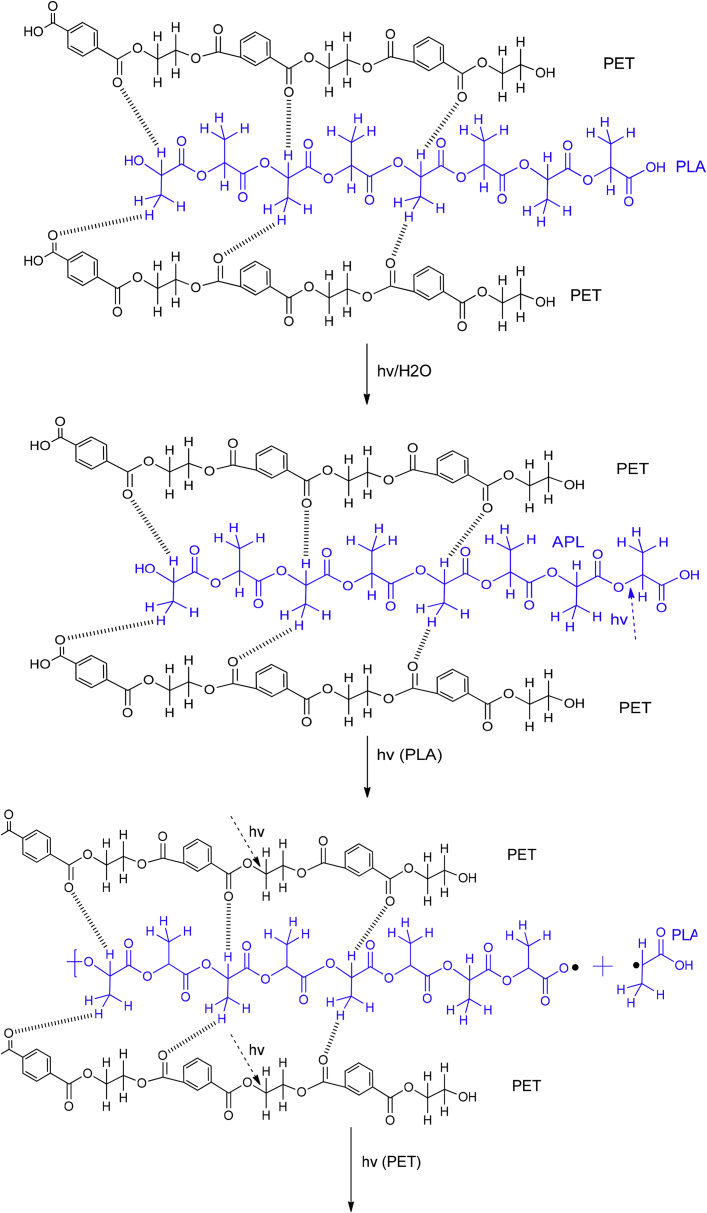

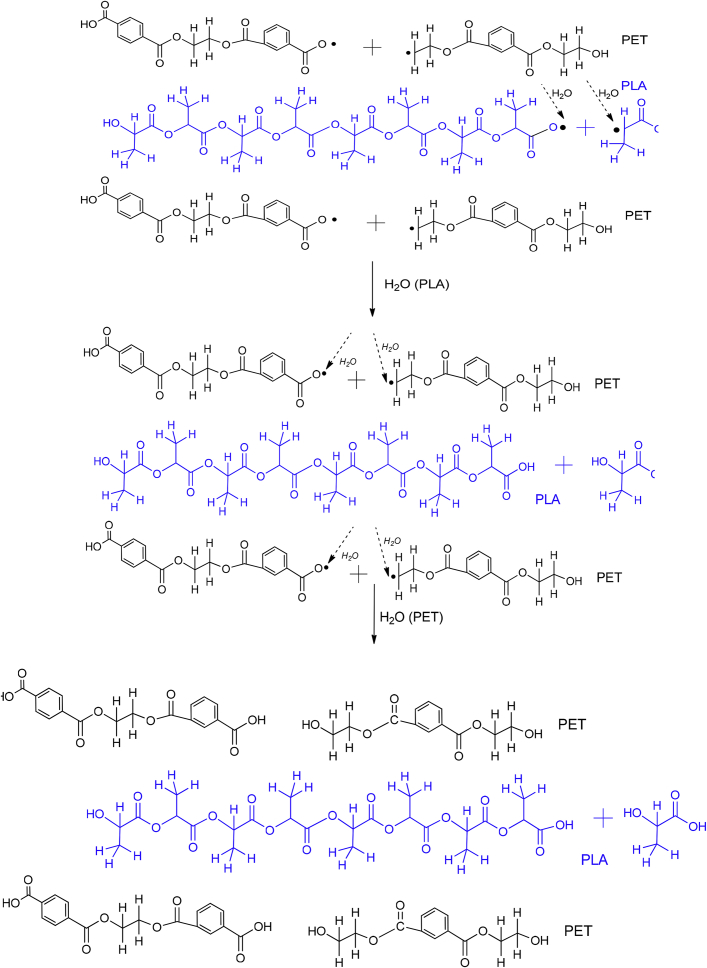
Proposed degradation mechanism of Poly(ethylene terephtalate)(PET) modified with poly(lactic acid) (PLA).

**Fig. 4:**
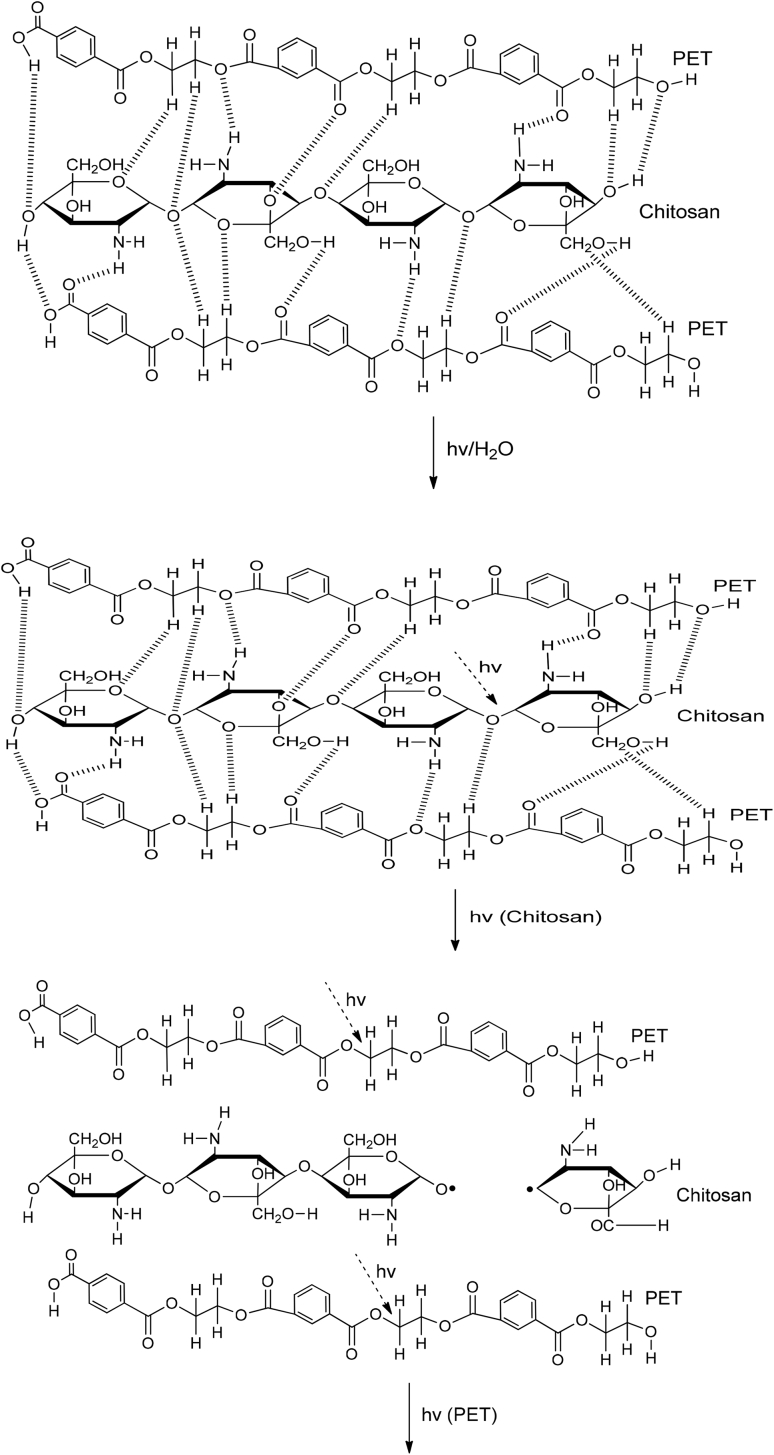

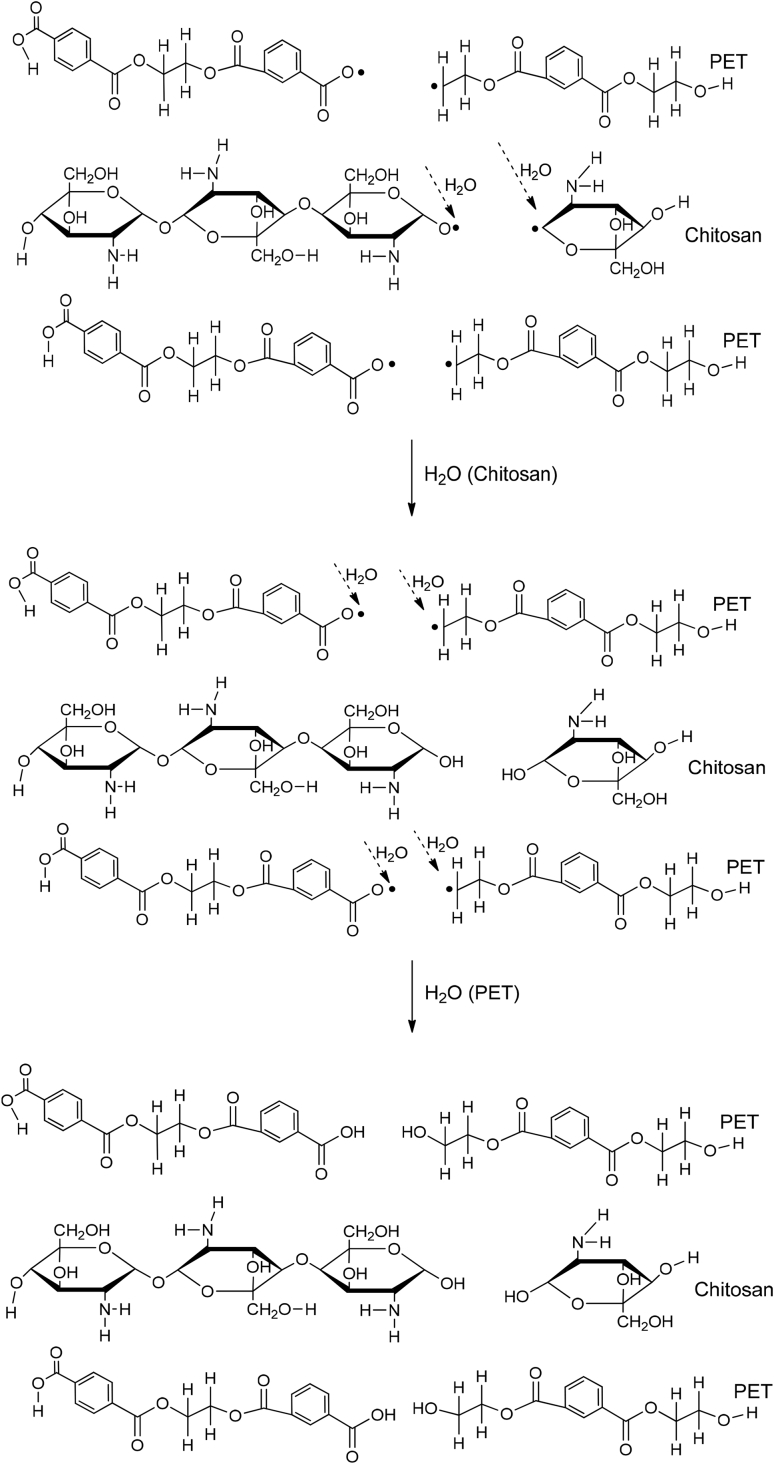
Proposed degradation mechanism of Poly(ethylene terephtalate)(PET) modified with chitosan.

**Fig. 5:**
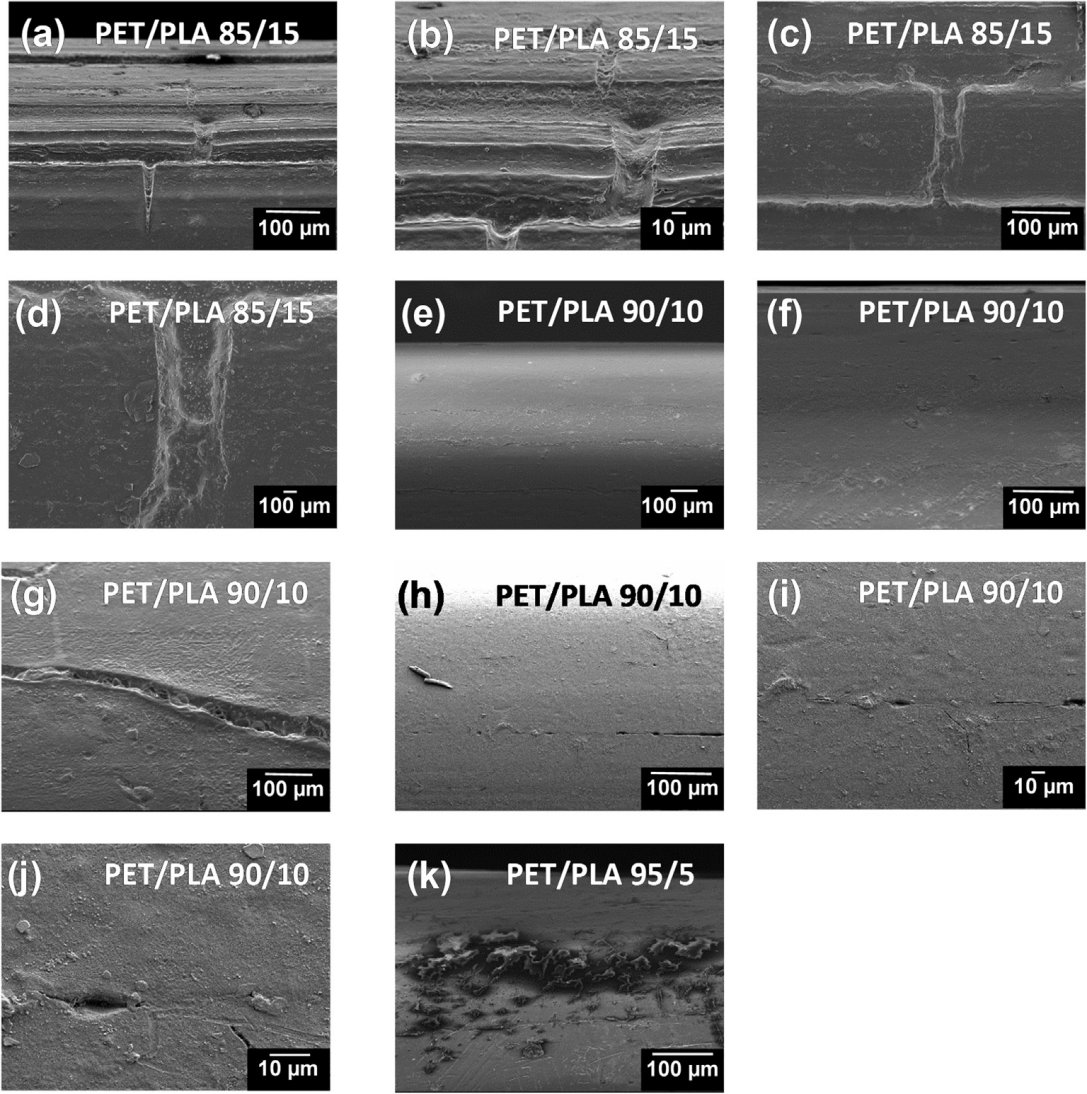
SEM micrographs showing the damage provoked after 900 h of accelerated weathering in 95/5, 90/10 and 85/15 ratio of PET/PLA blends.

**Fig. 6:**
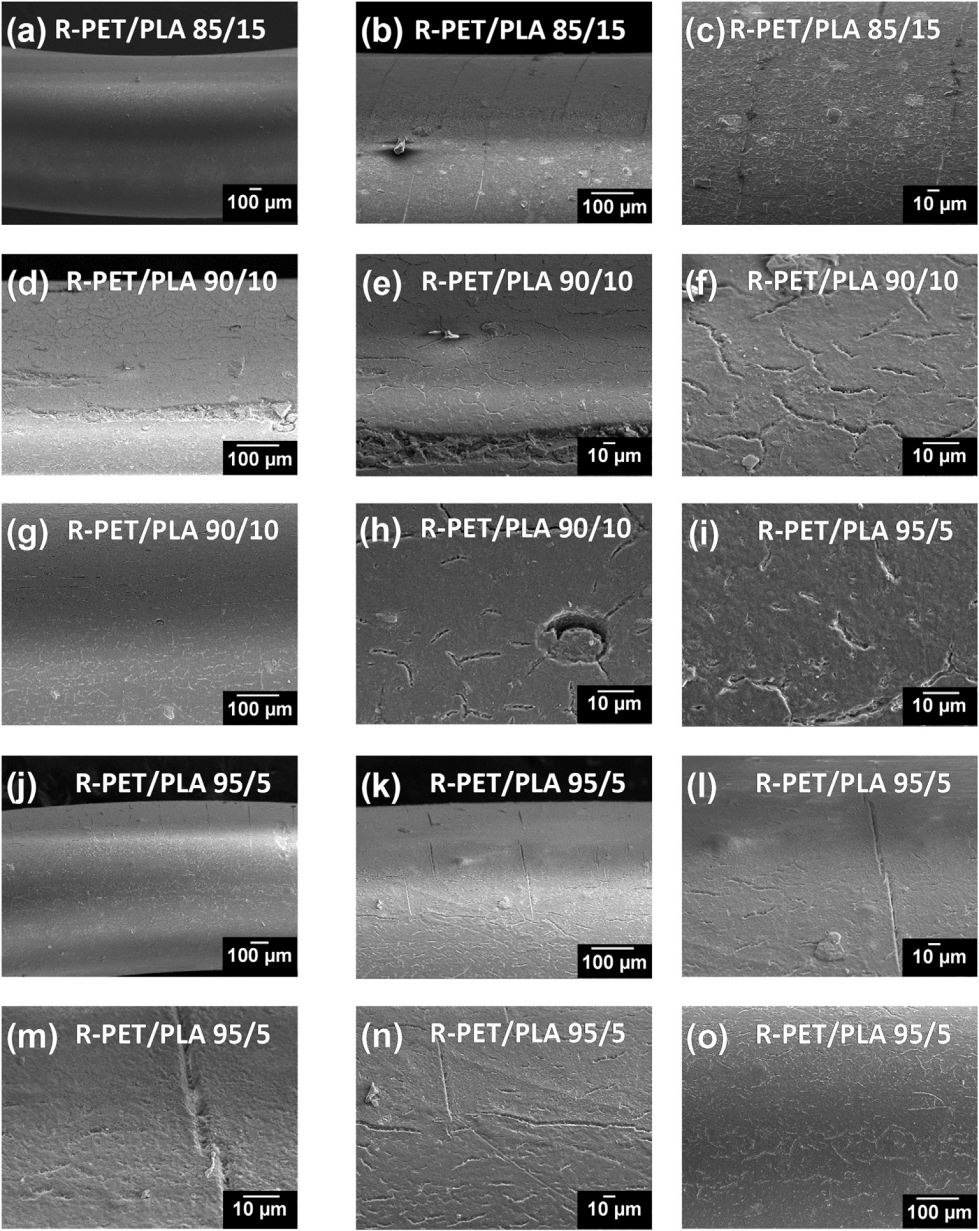
SEM micrographs showing the damage provoked after 900 h of accelerated weathering in 95/5, 90/10 and 85/15 ratio of R-PET/PLA blends.

**Fig. 7:**
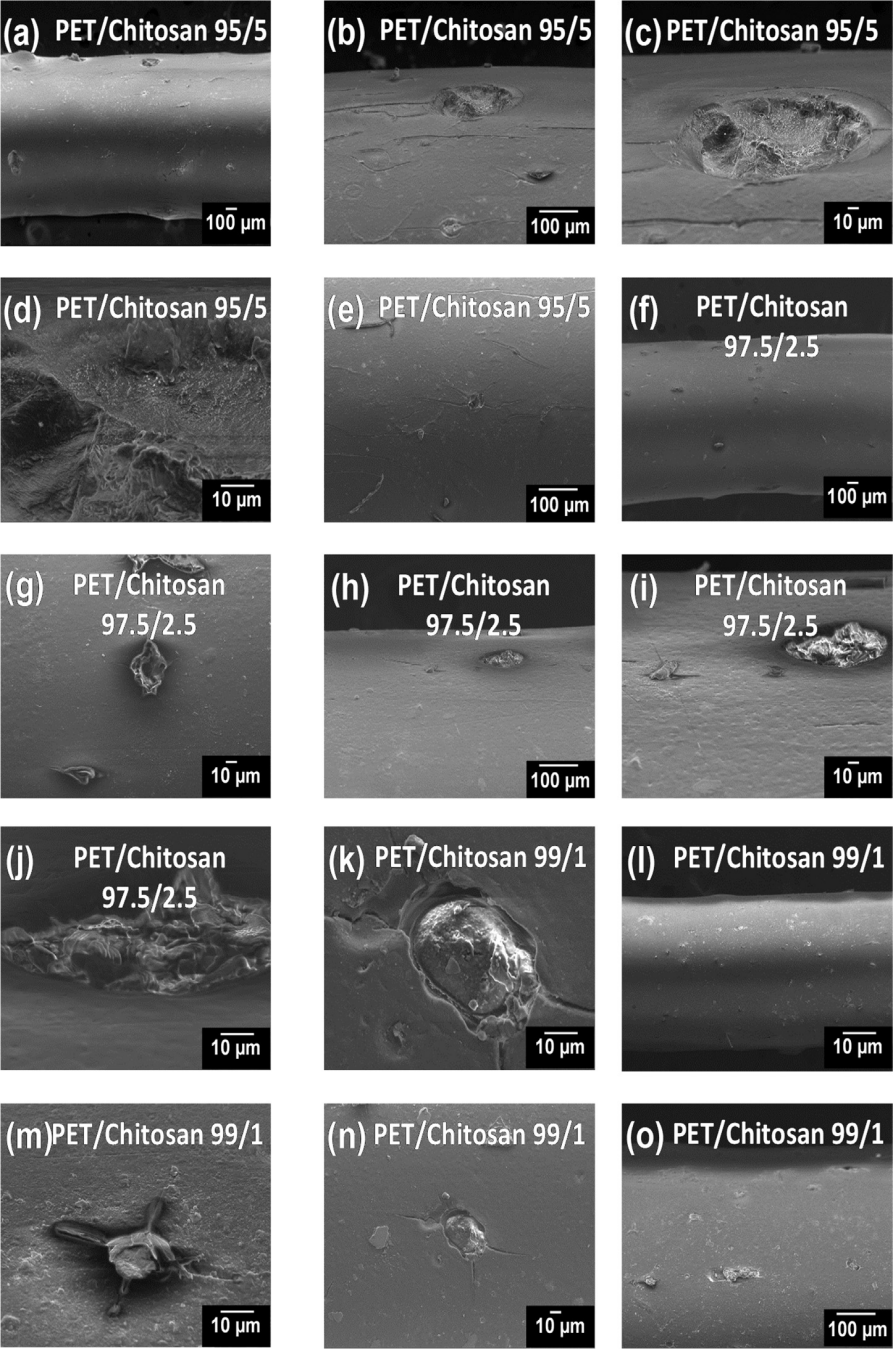
SEM micrographs showing the damage provoked by 900 h of accelerated weathering of 95/5, 97.5/2.5 and 99/1 PET/Chitosan blends.

**Fig. 8:**
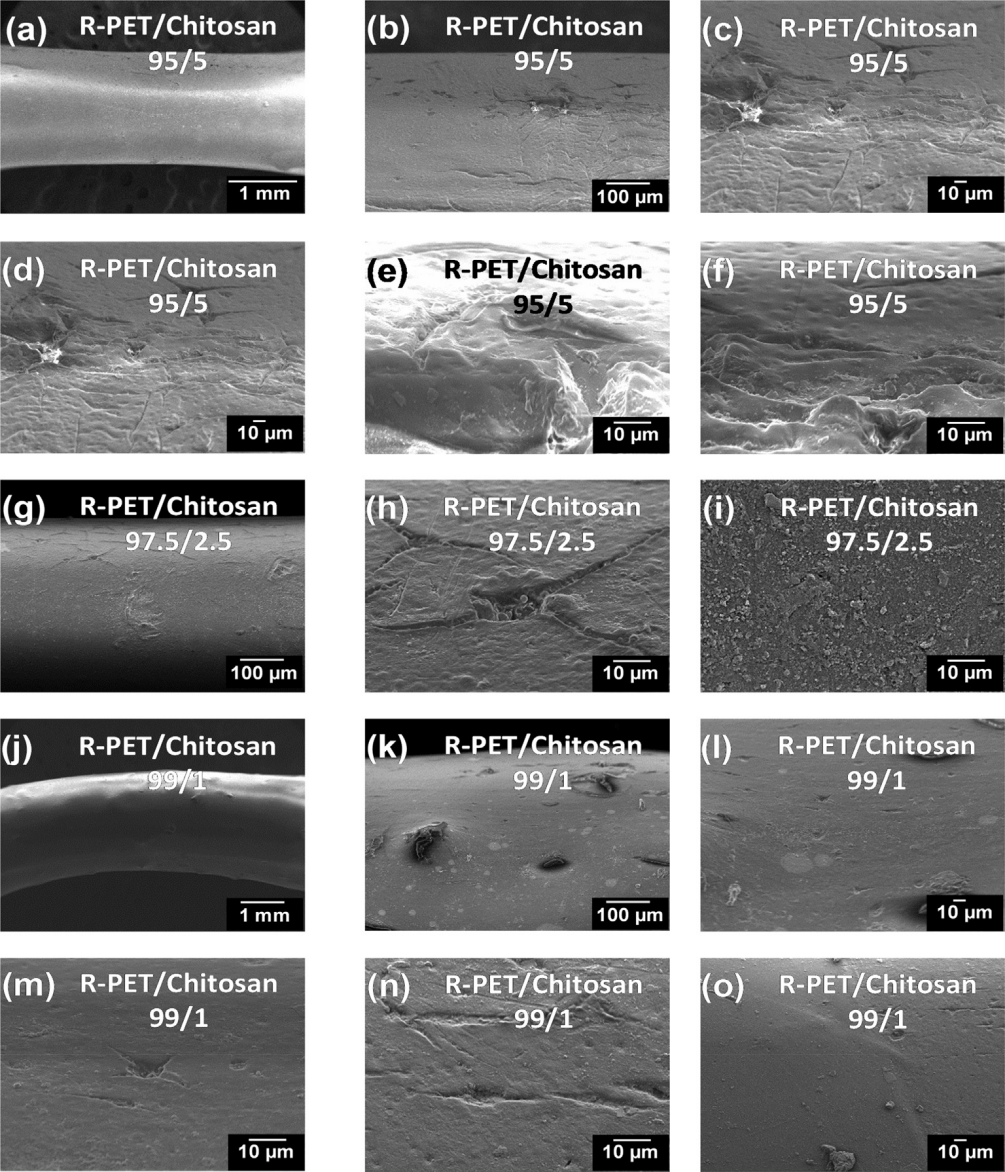
SEM micrographs showing the damage provoked by 900 h of accelerated weathering of 95/5, 97.5/2.5 and 99/1 R-PET/Chitosan blends.

PET and R-PET modified with either PLA or chitosan are compatible due to their physical interactions, which can be between the hydrogen bonding of each phase. These interactions are represented in Fig. 3, Fig. 4. The mechanism was proposed based on the Fourier Transform Infrared Spectroscopy (FT-IR) results reported in Ref. [1]. Also, the degradation and the byproducts provoked by UV light are shown on them. Free radicals and hydrolysis are created when PET/PLA and PET/chitosan blends are degraded under ambient conditions. Complementary SEM micrographs to those reported in Ref. [1] of weathered blends after 900 h are shown in Fig. 5, Fig. 6, Fig. 7, Fig. 8.

## 2 Experimental design, materials, and methods

A first set of experiments was stablished by using PET pellets (CLEARTUF®-MAX2, lot no. 1008–03219) provided by M&G Polymers Company. A second set of experiments was done using recycled PET (R-PET) obtained from discarded bottles after they were washed, dried and cut into flakes. PET and R-PET were dried at 60 °C during 24 h in an oven (Thermolyne). 5, 10 and 15 wt% of PLA and 1, 2.5 and 5 wt% of chitosan were hand mixed, processed to obtain filaments in a single-screw extruder (C.W. Brabender) with L/D ratio of 25:1 and four heating zones: feeding (225 °C), compression (237.5 °C), distribution (260 °C), and the extrusion die (225 °C) [1].

To study the type of fractured surface, these filaments were prepared and fractured in an Instron universal testing machine (Model 5944) at a crosshead speed of 20 mm/min by using a load cell of 2 kN. Before acquiring the micrographs in Scanning Electron Microscopy (SEM) equipment, they were dried at 40 °C for 24 h. Then, filaments were coated with an Au–Pd thin film on a Quorum Q150T ES sputter coater system. SEM images were taken in a JEOL equipment (JSM-6300 model) equipped with a termoemission cathode based on Tungsten (W) at a vacuum of 10^−4^ Pa while using the X-vision system (computer software) with an image capture of 2048 × 1536 × 8bit. The fractured surface of PET/PLA, R-PET/PLA, PET/Chitosan and R-PET/Chitosan was analyzed by acquiring SEM images from the detected secondary electrons of the filaments at low voltage of accelerating (15 kV) and magnifications of 75×.

An Atomic Force Microscope (AFM), EasyScan 2 AFM/STM model (Nanosurf) equipped with a scanner with maximum values in xy:z directions of 10:2, 79:14; 110:22 μm, was used to estimate the roughness data and the topographical features of PET/PLA, R-PET/PLA, PET/Chitosan, R-PET/Chitosan selected samples. All AFM scans were acquired under ambient conditions in tapping mode using areas of 1 μm × 1 μm using silicon cantilevers with nominal probe curvature radius of 10 nm and a nominal force of 20 nN. The images were acquired at a resolution of 512 × 512 points. The AFM data were processed with the WSxM 5.0 Develop 2.0 software (Nanotec, Inc.) to acquire the roughness values (RMS and Ra).

The filaments were subjected to accelerated weathering test as described in Ref. [1] and their morphologies of degraded surfaces after 900 h of exposure, were acquired using a JEOL-JSM-6500 F thermal Field emission Scanning Microscopy (FE-SEM) with a FE source of Schottky type by using the “Analysis Station” software and secondary electrons to acquire the images. The accelerating voltage used in this study was 7.0 kV at 10^−4^ Pa of vacuum.
