# Supplementary material for: Data supporting the morphological/topographical properties and the degradability on PET/PLA and PET/chitosan blends
Source: Data Brief. 2019 May 24;25:104012. doi: 10.1016/j.dib.2019.104012 (PMC6552015; doi:10.1016/j.dib.2019.104012)
Supplement: Multimedia component 1 [file mmc1.pdf]

AUTHORS DECLARATION

We wish to confirm that there are no known conflicts of interest associated with this publication and there has been no significant financial support for this work that could have influenced its outcome.

We confirm that the manuscript has been read and approved by all named authors and that there are no other persons who satisfied the criteria for authorship but are not listed. We further confirm that the order of authors listed in the manuscript has been approved by all lof us.

We confirm that we have given due consideration to the protection of intellectual property associated with this work and that there are impediments to publication, including the timing of publication, with respect to intellectual property. In so doing we confirm that we have followed the regulations of our instructions concerning intellectual property.

We understand that the Corresponding Authors are the sole contact for the Editorial process (including editorial manager and direct communications with the office). He/she is responsible for communicating with the other authors about progress, submission of revisions and final approval of proofs. We confirm that we have provided a current, correct email address which is accessible by the corresponding author and which has been to accept email from.

Aidé Minerva Torres-Huerta	atorresh@ipn.mx
Diana. Palma-Ramírez	d.palma.rmz@gmail.com
Miguel Antonio Domínguez-Crespo	adcrespo2000@yahoo.com.mx
Deyanira Del Angel-López	yaderani@hotmail.com
A.I. Flores-Vela	aforesv@ipn.mx
Daniel de la Fuente	delafuente@cenim.csic.es

Scanned with CamScanner
